# Hermansky-Pudlak syndrome-2 alters mitochondrial homeostasis in the alveolar epithelium of the lung

**DOI:** 10.1186/s12931-021-01640-z

**Published:** 2021-02-08

**Authors:** Karina Cuevas-Mora, Willy Roque, Hoora Shaghaghi, Bernadette R. Gochuico, Ivan O. Rosas, Ross Summer, Freddy Romero

**Affiliations:** 1 Department of Medicine, Division of Pulmonary, Allergy and Critical Care and the Center for Translational Medicine, The Jane & Leonard Korman Respiratory Institute, Philadelphia, PA USA; 2 Department of Medicine, Rutgers – New Jersey Medical School, 185 S Orange Ave, Newark, NJ 07103 USA; 3 Medical Genetics Branch, National Human Genome Research Institute, National Institutes of Health, Bethesda, MD USA; 4 Pulmonary, Critical Care and Sleep Medicine, Baylor College of Medicine, Houston, TX USA

## Abstract

**Background:**

Mitochondrial dysfunction has emerged as an important player in the pathogenesis of idiopathic pulmonary fibrosis (IPF), a common cause of idiopathic interstitial lung disease in adults. Hermansky-Pudlak syndrome (HPS) is a rare autosomal recessive disorder that causes a similar type of pulmonary fibrosis in younger adults, although the role of mitochondrial dysfunction in this condition is not understood.

**Methods:**

We performed a detailed characterization of mitochondrial structure and function in lung tissues and alveolar epithelial cells deficient in the adaptor protein complex 3 beta 1 (*Ap3b1*) subunit, the gene responsible for causing subtype 2 of HPS (HPS-2).

**Results:**

We observed widespread changes in mitochondrial homeostasis in HPS-2 cells, including the acquisition of abnormally shaped mitochondria, with reduced number of cristae, and markedly reduced activity of the electron transport chain and the tricarboxylic acid cycle. We also found that mitochondrial redox imbalance and activity of the mitochondrial unfolded protein response were dysregulated in HPS-2 cells and this associated with various other changes that appeared to be compensatory to mitochondrial dysfunction. This included an increase in glycolytic activity, an upregulation in the expression of mitochondrial biogenesis factors and enhanced activation of the energy-conserving enzyme AMP-activated protein kinase.

**Conclusion:**

In summary, our findings indicate that mitochondrial function is dramatically altered in HPS-2 lung tissues, suggesting dysfunction of this organelle might be a driver of HPS lung disease.

## Introduction

Idiopathic pulmonary fibrosis (IPF) is a highly aggressive form of lung disease that develops in older individuals and is generally refractory to existing pharmacological therapies [1–5]. Although the underlying mechanisms leading to IPF are not entirely understood, mitochondrial dysfunction has emerged as an important pathogenic factor in this disease [6–10]. For example, Bueno et al. showed that large, dysmorphic mitochondria accumulate in the distal pulmonary epithelium of IPF patients; a phenomenon linked to defective mitophagy from reduced PTEN-induced putative kinase 1 (PINK1) activity [6]. Moreover, others have reported additional mitochondrial derangements in lungs of IPF patients, such as an increase in mitochondrial ROS production, altered bioenergetics and reduced organelle biogenesis [8]. Indeed, Yu et al. demonstrated that pulmonary fibrosis in IPF animal models could be blunted by augmenting mitochondria biogenesis with the hormone thyroxine, suggesting a role for mitochondrial-directed therapies in the treatment of this disease [8].

Patients with the Hermansky-Pudlak syndrome (HPS) develop a form of fibrotic lung disease that closely resembles IPF [11, 12]. HPS is a rare autosomal recessive disorder that affects approximately 1–9 per 1,000,000 individuals and results from bi-allelic variants in one of several different lysosomal trafficking genes [13]. To date, 11 different genetic types of HPS have been reported, with each HPS gene encoding a unique protein subunit in the Biogenesis of Lysosome-related Organelles Complex-1, -2, or -3 or the adaptor protein complex 3 [11, 14–18]. While all patients with HPS develop some degree of oculocutaneous albinism and platelet dysfunction, only patients with HPS-1, HPS-2 or HPS-4 develop pulmonary fibrosis [11, 12, 15, 19]. Importantly, pulmonary fibrosis in HPS is often a lethal complication, emphasizing the need to understand its pathogenic mechanisms.

Although IPF and HPS are believed to arise from different mechanisms, a growing body of evidence has suggested interesting parallels between these conditions. For example, similar histopathological changes can be detected in the lungs of both IPF and HPS patients [11]. Moreover, it is generally believed that alveolar epithelial dysfunction underlies the pathogenesis of both diseases [6, 20–22]. Additionally, we, and others, have uncovered that many of the same inflammatory and tissue-remodeling factors are dysregulated in the blood and lungs of IPF and HPS patients [23, 24]. With these findings in mind, we hypothesized that mechanistic advances in the IPF field could provide a roadmap for better understanding the processes leading to epithelial dysfunction in HPS. In this study, our primary objective was to determine whether HPS alters mitochondrial function in ways similar to the lung of IPF patients and explore whether these changes are associated with other metabolic perturbations. To achieve these objectives, we employed two models of the genetic subtype 2 of HPS. This included a well-characterized mouse model and a novel alveolar epithelial cell line generated by CRISPR-Cas9 gene-editing techniques. Notably, the use of CRISPR-Cas9 gene-editing techniques to model HPS in lung cells has been used by other laboratories, illustrating the power of this technology [25].

## Methods

### Animals

C57BL/6 J and HPS mice were purchased from the Jackson Laboratories (Bar Harbor, ME) and housed in a pathogen-free animal facility at Thomas Jefferson University. To model HPS, we selected HPS-2 mice, which harbor a homozygous mutation in the *Ap3b1* gene [20, 26]. Prior to performing studies, all animal protocols were approved by the Institutional Animal Care and Use Committees at Thomas Jefferson University.

### Electron microscopy

Mitochondrial structure was evaluated in paraformaldehyde and glutaraldehyde fixed lung tissues from C57BL/6J and HPS-2 mice. After fixation, tissues were processed and sectioned and then imaged with an electron microscope per published protocols [27].

### Mitochondrial isolation

Mitochondria were isolated from lung digests using a commercially available kit (Miltenyi Biotec, Auburn, CA) according to manufacturer’s instructions. In brief, approximately 250 mg of tissue was excised from the lung and minced into smaller fragments using a razor blade digestion method and a GentleMACS Tissue Dissociator (Miltenyi Biotec).

### Measurement of mitochondrial complex I and IV activities

Mitochondrial Complex I and Complex IV activities (Abcam, Cambridge, MA) were measured using a commercially available kit per manufacturer’s instructions.

### Mitochondrial ROS assessments

Mitochondrial ROS levels were measured using Mito-Sox Red dye (Invitrogen, Carlsbad, CA) per published protocols [28]. In brief, cells were exposed to Mito-Sox Red dye (5 μM) for 10 min at 37 C and fluorescence intensity was quantified with a microplate reader.

### Isolation of primary alveolar epithelial type II cells

Primary alveolar epithelial type II (AE2) cells were isolated from the mouse lung as previously described [29]. In brief, dispase (BD Bioscience, San Jose, CA) and low melting agarose (Bio-Rad; 1%, 0.5 ml) were sequentially instilled into the airways before extracting lungs *en bloc* and immediately placing tissues on ice to harden agarose. Next, lung tissues underwent mechanical digestion using the Gentle Macs tissue dissociator (Miltenyi Biotec). Purified preparations of AE2 cells were recovered using a two-step process that first removed CD45-expressing immune cells and then positively selected lung epithelial cells with an anti-Ep-CAM antibody [29].

### Aconitase and fumarase activity

Freshly isolated cells were suspended in 0.5 ml of buffer containing 1 mM Tris–HCL (pH 7.4). Aconitase and fumarase activity were measured according to manufacturer’s instructions (Abcam). Aconitase measures the conversion of citrate to isocitrate, whereas fumarase measures the conversion of fumarate to malate. Enzymatic activity was quantified based on absorbance at 450 nm at 37 °C and results were normalized to protein concentration.

### Lactate assay

Lactate was measured in the soluble fraction according to manufacturer’s instructions (BioVision,). In the lactate assay kit, lactate is oxidized by lactate dehydrogenase to generate a product that interacts with a probe to produce color at an absorbance of 450 nm.

### Cell culture and CRISPR/Cas-based knockout of *AP3b1* in MLE15 cells

HPS-2 lung epithelial cells were generated by our laboratories using the mouse lung epithelial 15 cell line, a strategy used by other labs to generate HPS lung epithelial cells [25]. CRISPR-Cas9 editing of *Ap3b1* was performed at Thermo Fisher Scientific (St. Louis, MO).

### Oxygen consumption measurements

Oxygen consumption rate (OCR) was measured using the Seahorse XFP6 Bioanalyzer (Seahorse Bioscience, Billerica, MA) as previously described [28, 30]. In brief, cells were seeded at a concentration of 30,000 cells/well on XFP cell plates 24 h prior to the initiation of studies. The concentration of FCCP (also known as rifluoromethoxy carbonylcyanide phenylhydrazone), antimycin A, and rotenone were 2, 0.75, and 1 μM, respectively. To measure inner mitochondria membrane complex activity, OCR measurements were performed on cells exposed to the XF plasma membrane permeabilizer reagent. The concentration of permeabilizer reagent was determined after assessing the effects of different concentrations on cell viability. Pyruvate (10 mM) and malate (1 mM) served as a substrate, in addition to ADP (4 mM). The final concentrations of injected compounds were as follows: port A, 2 μM rotenone; port B, 10 mM succinate; port C, 4 μM antimycin A; port D, 100 mM ascorbate plus 1 mM TMPD (tetramethyl-p-phenylenediamine). The protocol and algorithm for our XF-PMP assay were designed using wave 2.4 software.

### Mitochondrial DNA copy number

Nuclear and mitochondrial (mt) DNA copy number were assessed by qPCR as previously described. In brief, genomic DNA was isolated using the Qiagen Genomic-Tip 20G and Qiagen DNA Buffer kit. PCR was performed to amplify mtDNA (mtND1) and nuclear DNA (β-globin). Primers sequences are included in Additional file 1: Table S1. DNA was quantified using Pico-Green (Thermo Fisher Scientific) and a microplate fluorescent reader (PerkinElmer, Waltham, MA.) at excitation and emission wavelengths of 485 and 530 nm, respectively. Relative amounts of mitochondrial DNA to β-globin genomic sequence was calculated according to 2−^ΔΔCt^ method.

### RNA isolation and analysis

Gene transcript levels were quantified by real-time PCR as previously described [31]. In brief, RNA was isolated using RNeasy Mini-Kit (QIAGEN, Valencia, CA). All reactions were performed with 1 μM of forward and reverse primers along with SYBR Green I GoTaq qPCR Master Mix (Promega, Madison, WI). A summary of primer sets manufactured by Integrated DNA Technologies can be found in Additional file 1: Table S1. Primer sets were amplified using protocols previously described. All values were normalized to a control gene such as *18S*.

### Western blot analysis

Protein concentration was determined by Pierce™ BCA assay kit (Thermo Scientific). Aliquots of protein lysates were transferred onto nitrocellulose membranes and then blocked with the Odyssey Blocking Buffer (Li-Cor Biosciences, Lincoln, NE) for 1 h at RT. This was followed by an incubation step with a specific polyclonal rabbit primary antibody directed against HSP10, mtHSP70, LONP1, CLPP, AMPK, p-AMK^Thr172^, SIRT3, PGC1α, NRF1, HTRA/OMI, and β-actin (Sigma-Aldrich, St. Louis, MO). Next, membranes were incubated in a solution containing a donkey anti-rabbit or anti-mouse antibody (Li-Cor Biosciences). After three sequential washes with PBS, immunoblots were visualized using the Odyssey infrared imaging system (Li-Cor Biosciences).

### Statistical analysis

Statistics were performed using GraphPad Prism 8.0 software. Comparisons between two groups were done using an unpaired Student’s t-test. Statistical significance was achieved when *P* < 0.05 at 95% confidence interval.

## Results

### HPS-2 alters mitochondrial function and drives metabolic reprogramming in the alveolar epithelium

**Fig. 1:**
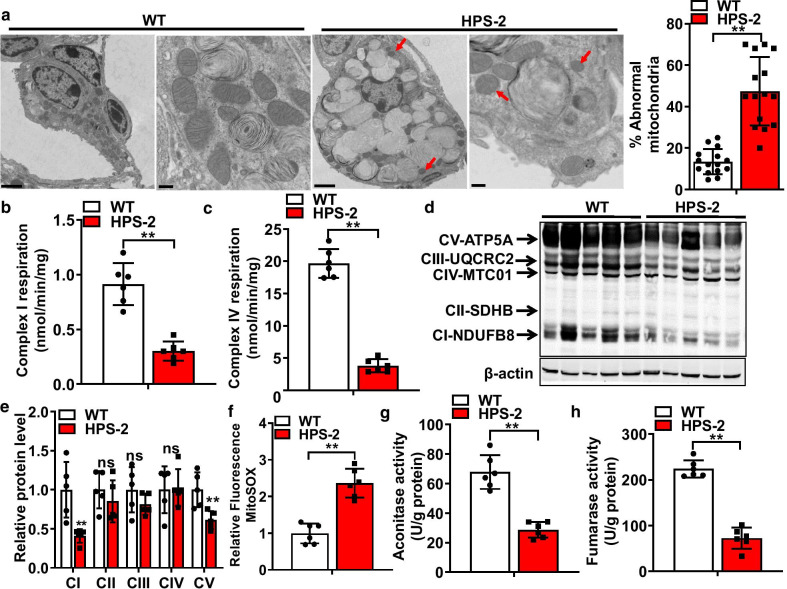
HPS-2 alters mitochondrial structure and function in the alveolar epithelium of the lung. **a** Representative TEM images of AE2 cells from lungs of WT and HPS-2 mice. Mitochondria from HPS-2 mice show smaller mitochondria (red arrow) with reduced number of cristae. Bar graph depicts number of abnormally-shaped mitochondria per AE2 cell (n = 15 per group). **b**, **c** Mitochondrial Complex I and IV activity in mitochondrial isolates from lungs of WT and HPS-2 mice (n = 6, each group). **d** Levels of ETC protein subunits in lungs of WT and HPS-2 mice. **e** Densitometry analysis of ETC protein complexes. **f** Mitochondrial ROS levels in alveolar epithelial (AE2) cells from lungs of WT and HPS-2 mice (n = 5, each group). **g**, **h** Aconitase and fumarase activity in AE2 cells from lungs of WT and HPS-2 mice (n = 6, each group). Statistical significance was assessed by Student’s *t*-test. *P < 0.05, **P < 0.01 vs. control group. Densitometry analyses (bar graphs) are representative n = 5 or more mouse specimens

**Fig. 2:**
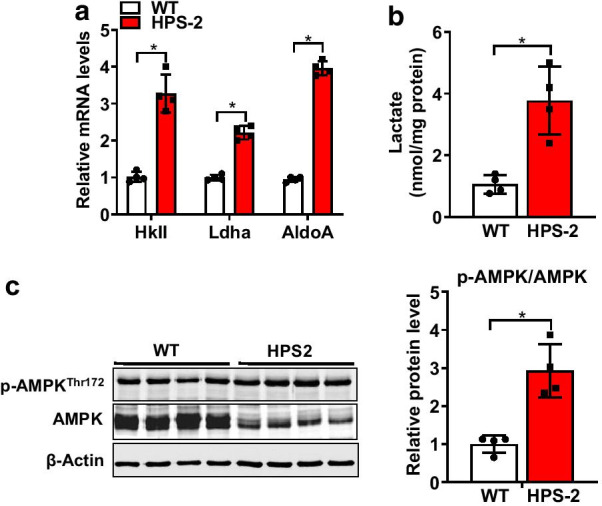
HPS-2 induces metabolic reprograming in the mouse lung. **a** Transcript levels for hexokinase II and lactic dehydrogenase in isolated AE2 cells from WT and HPS-2 mice (n = 4, each group). **b** Lactate levels in lungs of WT and HPS-2 mice (n = 4, each group). **c** Immunoblot for total and phosphorylated AMPK levels in lungs of WT and HPS-2 mice. Densitometry measurements shown on the right. Student’s *t*-test. *P < 0.05 vs. control group. Densitometry analyses (bar graphs) are representative n = 4 or more mouse specimens

To begin to assess the effects of HPS on mitochondrial function in the lung, we performed ultrastructural analyses to examine the morphology of mitochondria in AE2 cells from the lungs of C57B6/6J (WT) and HPS-2 mice. Our decision to select HPS-2 mice was based on the understanding that mutations in *Ap3b1* are linked to HPS pulmonary fibrosis in humans and enhance the sensitivity to bleomycin in mice [20, 26, 32]. As shown in Fig. 1, we observed that mitochondria in AE2 cells of HPS-2 mice were smaller; more rounded and displayed fewer cristae than AE2 cells in control mice, although mitochondrial number was difficult to assess due to the massive size of lamellar bodies in the cytoplasm of HPS-2 cells. Moreover, these structural changes associated with a nearly twofold decrease in oxidation of NADH to NAD^+^ at Complex I and activity of cytochrome c oxidase at Complex IV (Fig. 1b, c) as well as a significant reduction in protein levels for select electron transport chain (ETC) protein complexes (Fig. 1d, e), namely complex I and IV. We also observed that HPS AE2 cells exhibited a significant increase in mitochondrial ROS levels and a marked reduction in activity of the TCA cycle, as measured by aconitase and fumarase activities (Fig. 1g, h). This was also associated with various other metabolic perturbations that appeared compensatory to mitochondrial dysfunction, such as an increase in transcript levels for lactic dehydrogenase A (Ldha), hexokinase II (HkII) and aldolase A (AldoA) and an increase in lactic acid levels (Fig. 2a, b). We also found that phosphorylation of the energy-conserving enzyme 5′-AMP-activated protein kinase (AMPK) was increased in the lungs of HPS-2 mice (Fig. 2c), supporting the notion that cells are trying to compensate for mitochondrial dysfunction.

**Fig. 3:**
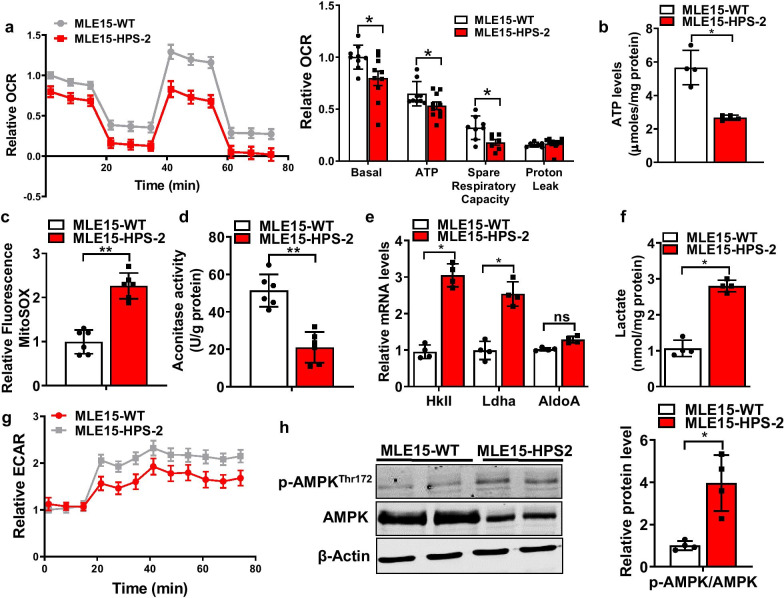
Mitochondrial function is impaired in the HPS-2 alveolar epithelium. **a** Mitochondrial oxygen consumption in control and HPS-2 mouse lung epithelial (MLE) cells (n = 3, each group). **b** Total ATP levels in control and HPS-2-MLE15 cells (n = 4, each group). **c** Mitochondrial ROS levels in control and HPS-2-MLE15 cells (n = 6, each group). **d** Aconitase activity in control and HPS-2-MLE15 cells (n = 6, each group). **e**–**g** Glycolytic activity in control and HPS-2-MLE15 cells as measured by transcript levels for hexokinase and lactate dehydrogenase, lactate levels and extracellular acidification rate (ECAR) (n = 3–4, each group). **h** Immunoblot for total and phosphorylated AMPK levels in control and HPS-2- MLE15 cells (n = 4, each group). Densitometry measurements shown on the right. Student’s *t*-test. *P < 0.05, **P < 0.01 vs. control group

Because primary AE2 cells do not tolerate extensive in vitro manipulations, we next developed an alternative model to study the effects of HPS on mitochondrial homeostasis. Using a CRISPR-Cas9 gene-editing approach, we developed HPS-2 lung epithelial cells using the mouse lung epithelial 15 cell line, a line commonly used to model AE2 cells in culture and previously used by other groups to model HPS cells in vitro [25, 33]. Consistent with findings in primary mouse tissues, we found that mitochondrial function was significantly altered in HPS-2 MLE15 cells. This included a reduction in mitochondrial oxygen consumption rate (OCR), and a marked decrease in total ATP levels (Fig. 3a, b). Moreover, we found this associated with an increase in mitochondrial ROS levels, as measured by the MitoSox assay, and markedly reduced aconitase activity (Fig. 3c, d). Additionally, like primary mouse lung epithelial cells, we discovered other metabolic changes in MLE15-HPS-2 cells, including an increase in glycolytic activity (Fig. 3e–g) and enhanced phosphorylation of AMPK (Fig. 3h).

### HPS-2 leads to hyperactivation of the UPR^mt^ in lung epithelium

**Fig. 4:**
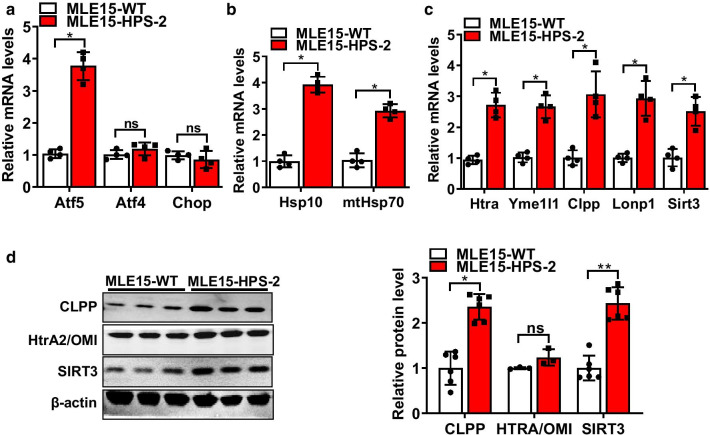
The mitochondrial unfolded protein response is increased in HPS-2 alveolar epithelial cells. **a** Transcript levels for activating transcription factor 4, 5 and C/EBP Homologous Protein in control and HPS-2-MLE15 cells (n = 4, each group). **b**, **c** Transcript levels for heat shock protein (HSP) 10 and HSP70, serine protease Htra2, the mitochondrial proteases yeast mitochondrial AAA metalloprotease (Yme1el), caseinolytic Mitochondrial Matrix Peptidase Proteolytic Subunit (CLPP) and long protease 1 (Lonp1) and Sirtuin 3 (Sirt3) in control and HPS-2-MLE15 cells (n = 4–5, each group). **d** Western blot for CLPP, HtrA2 and SIRT3 in control and HPS2-MLE15 cells (n = 3–6, each group). Beta actin was used as a loading control. Densitometry measurements are shown together. Student’s *t*-test. *P < 0.05, **P < 0.01 vs. control group

**Fig. 5:**
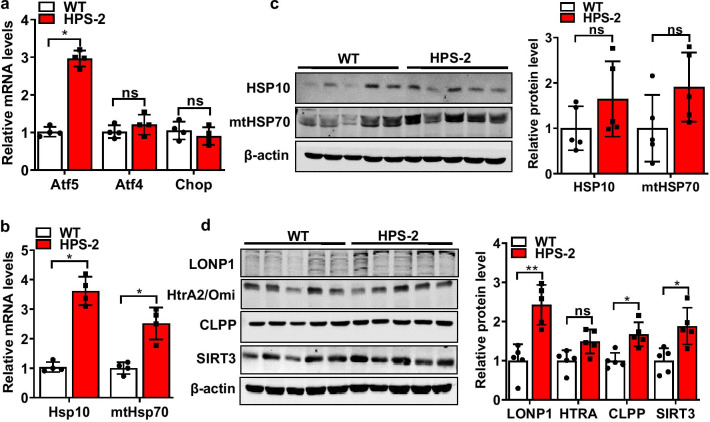
Mitochondrial unfolded protein response is upregulated in the lungs of HPS-2 mice. **a** Transcript levels for Atf5, Atf4 and Chop in lungs of WT and HPS-2 mice (n = 4, each group). **b** Transcript levels HSP10, mtHSP70 (n = 4, each group). **c** Western blot for HSP10 and mtHSP70 in lungs of WT and HPS-2 mice (n = 5, each group). Beta actin was used as a loading control. Densitometry measurements are shown together. **d** Western blot for LONP1, HtrA2, CLPP and SIRT3 in lungs of WT and HPS-2 mice (n = 5, each group). Densitometry measurements shown on the right. Student’s *t*-test. *P < 0.05 vs. control group. Densitometry analyses (bar graphs) are representative n = 5 or more mouse specimens

Under healthy conditions, mitochondrial homeostasis is maintained, in part, by activation of the mitochondrial unfolded protein response (UPR^mt^), a signaling pathway that functions to reduce excessive protein aggregation in mitochondria. In an attempt to understand why HPS causes mitochondrial dysfunction, we hypothesized that Ap3b1 deficiency might alter the function of this protective response. To test this, we compared levels of various UPR^mt^ mediators in control and HPS-2 cells. To our surprise, we found that transcript and proteins levels for nearly all mediators were increased relative to controls, indicating a hyperactivated UPR^mt^. This included ATF5, one of two major transcriptional regulators of UPR^mt^ genes, and various other downstream effector molecules, such as the chaperone proteins heat shock protein (hsp) 10 and mitochondrial HSP70 (mtHsp70). We also detected an increase in several enzymes critical to the maintenance of mitochondrial proteostasis, such as serine peptidase 2 (HtrA2/Omi), yeast mitochondrial AAA metalloprotease (Yme1el), caseinolytic mitochondrial matrix peptidase proteolytic subunit (Clpp), long protease 1 (Lonp1) and the deacetylase sirtuin-3 (Sirt3) (Fig. 4a–d). Additionally, similar directional changes in transcript levels for Atf5, Hsp10 and mtHsp70 and protein levels for LONP1, CLPP and SIRT3 (Fig. 5) were observed in the lungs of HPS-2 mice, illustrating the reproducibility of our findings across in vivo and in vitro model systems.

### HPS-2 activates mitochondrial biogenesis pathways in the alveolar epithelium

**Fig. 6:**
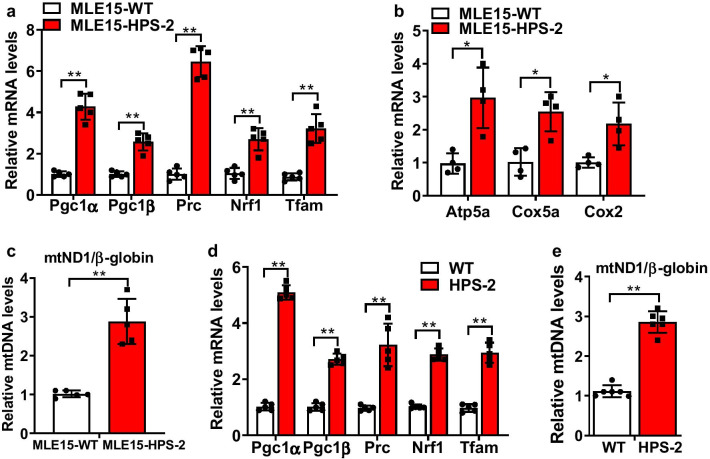
Mitochondrial biogenesis factors are increased in HPS-2 lung tissues. **a**, **b** Transcript levels for Pgc1α, Pgc1β, Prc, Nrf1, Tfam, Atp5a, Cox5a, and Cox2 in control and HPS-2-MLE15 cells (n = 4–5, each group). **c** Mitochondrial DNA content in control and HPS-2-MLE15 cells (n = 5, each group). **d** Transcript levels for Pgc1α, Pgc1β, Prc, Nrf1, Tfam in lungs of wild-type and HPS-2 mice (n = 5, each group). **e** Mitochondrial DNA content in lungs of wild-type and HPS-2 mice (n = 6, each group). Student’s *t*-test. *P < 0.05, **P < 0.01 vs. control group

Since HPS-2 led to hyperactivation of the UPR^mt^, we hypothesized that other homeostatic responses might also be affected in these cells. To test this, we next examined the effects of HPS-2 on factors controlling mitochondrial biogenesis. As shown in Fig. 6, we detected an increase in various mitochondrial biogenesis factors in HPS-2 cells. This included the transcriptional co-activators peroxisome proliferator-activated receptor γ coactivator 1 alpha (Pgc1α) and β (Pgc1β) as well as the protein Pgc related coactivator (Prc). We also found that levels of Nrf1 and Tfam were increased in HPS2-MLE15 cells (Fig. 6a) as were transcript levels for mitochondrial membrane ATP synthase (Atp5a), cytochrome c oxidase subunit 5a (Cox5a) and Cox2 subunit. This also associated with an increase in mitochondrial DNA (Fig. 6b, c), supporting the notion that organelle biogenesis was increased in these cells. Similar direction changes in biogenesis factors were also observed in the lung of HPS-2 mice, including the transcription regulators Pgc1α, Pgc1β, Prc, Nrf1 and Tfam (Figs. 6d) and the quantity of mtDNA (Fig. 6e). Together, these findings indicate that HPS-2 alters expression of mitochondrial biogenesis factors and further illustrates the profound effects that HPS has on mitochondrial homeostasis in lung cells.

## Discussion

**Fig. 7:**
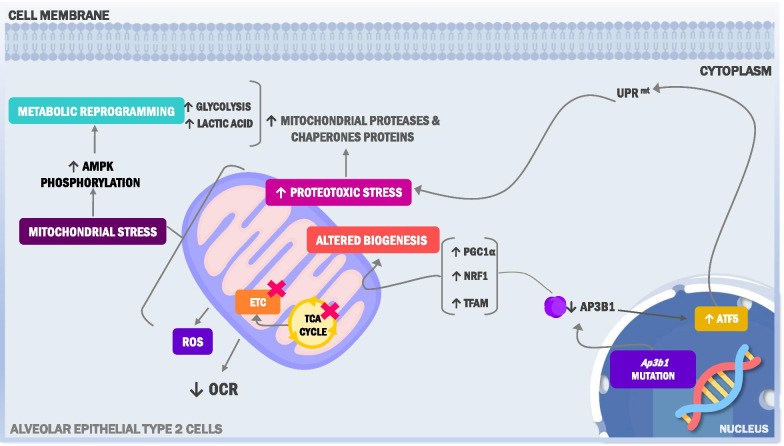
A cartoon depicting various aspects by which HPS-2 alters mitochondrial homeostasis in the alveolar epithelium. Ap3b1 mutation/deficiency alters multiple homeostatic processes in mitochondria including bioenergetics, TCA cycle, mitochondrial biogenesis, redox balance and proteotoxic stress responses. To date, the mechanisms leading to the wide array of cellular changes of HPS-2 alveolar epithelium remain unknown. *UPR*^*mt*^ mitochondrial unfolded protein response, *OCR* oxygen consumption rate; *ETC* electron transport chain; *TCA* tricarboxylic acid cycle; *ROS* reactive oxygen species, *PGC1α* peroxisome proliferator-activated receptor gamma coactivator 1-alpha; *NRF1* nuclear respiration factor 1; *TFAM* mitochondrial transcription factor A; *ATF5* activating transcription factor 5, *AP3B1* adaptor related protein complex 3 subunit beta 1

Mitochondrial dysfunction has emerged as an important pathogenic factor in the development of IPF, although its role in other fibrotic lung diseases is less well understood. In this study, we demonstrate that mitochondrial dysfunction is also a prominent feature of the lung in HPS and that HPS-2 mutations elicit a wide range of effects on this organelle, including altering bioenergetics and redox balance as well as affecting essential regulatory processes, such as the UPR^mt^ and production of biogenesis factors. Together, these findings open a new avenue of investigation in the HPS field and suggest that mitochondrial dysfunction might be a common factor contributing to the development of different types of fibrotic lung conditions. A cartoon depicting the various ways in which HPS-2 alters mitochondrial homeostasis is shown in Fig. 7.

Recent reports have shown that the alveolar epithelium in IPF undergoes dramatic metabolic reprogramming, and central to these changes is the reduction in mitochondrial oxygen consumption [8]. In this study, we found that mitochondrial oxygen consumption was also reduced in the HPS alveolar epithelium and this associated with various other derangements that appeared compensatory to mitochondrial dysfunction. Overall, these changes suggest that HPS and IPF cells are not just metabolically impaired but also have limited capacity to augment their energy production. We speculate these changes could have important consequences on the lung, such as altering its production of pulmonary surfactant or reducing its capacity to regenerate after injury. Perhaps, this also explains the why the IPF lung is so vulnerable to viruses and the HPS mouse lung is so sensitive to bleomycin [34–37].

In IPF, altered mitochondrial redox balance has been implicated in the pathogenesis of disease [2, 38, 39]. Reactive oxygen species are believed to drive fibrotic remodeling by inducing epithelial damage and promoting lung inflammation and myofibroblast activation. Although antioxidants have yet to show meaningful benefit in clinical trials, some have argued this may relate to the timing of administration, as antioxidants are often given in late stages of disease. Consistent with this line of reasoning, we detected an increase in mitochondrial ROS levels in “healthy” HPS mouse lung tissues. This included cells from the lungs of HPS mice that did not display evidence of fibrotic remodeling. Together, these findings support the notion that redox imbalances are an early manifestation of HPS and that antioxidants might serve as an early therapeutic intervention.

Another finding similar to IPF was the observation that markers of the UPR^mt^ were elevated in mouse HPS lung tissues [40]. Jiang et al. recently demonstrated a similar upregulation in markers of the UPR^mt^ in lungs of IPF patients [40]. Moreover, these investigators demonstrated that overexpression of ATF4 in the mouse alveolar epithelium, an essential mediator of the UPR^mt^ was sufficient to enhance fibrotic remodeling to bleomycin. Collectively, these findings suggest that activation of the UPR^mt^ serves as more than just a marker of mitochondrial dysfunction and may contribute directly to enhancing susceptibility to pulmonary fibrosis.

In addition to these similarities, we also identified several key differences between HPS and IPF tissues. For example, we found that expression of mitochondrial biogenesis factors was increased in HPS lung tissues, whereas several recent reports have described a reduction in mitochondrial biogenesis factors in the lungs of IPF patients [6, 8]. Moreover, Bueno et al. demonstrated that mitochondria size was increased in the alveolar epithelium of IPF patients whereas we found mitochondrial size was reduced in HPS tissues [6]. These findings suggest important mechanistic differences are likely to drive the development of mitochondrial dysfunction in HPS and IPF, and that elucidating these differences might help to uncover new disease-specific therapies.

Finally, we recognize our study has important limitations. For example, our investigations were performed exclusively with mouse tissues, making the relevance of our findings to humans less clear. Further, our studies focused on just a single HPS genetic subtype. Moreover, we recognize that HPS-2 is not the major subtype to cause pulmonary fibrosis in HPS patients, and that performing similar studies with HPS-1 and HPS-4 cells would have made our work more broadly applicable [11, 12]. That said, a recently published abstract by Kook et al. from the 2020 Experimental Biology Meeting reported that mitochondrial oxygen consumption was reduced in mouse HPS-1 alveolar epithelial cells [41], suggesting our findings are relevant to other HPS subtypes. Lastly, we recognize that our studies did not establish a mechanistic link between mitochondrial dysfunction and pulmonary fibrosis. However, we hope to establish this link in future investigations.

## Conclusion

In summary, our study is the first to demonstrate that HPS-2 alters mitochondria homeostasis in the lung and to suggest that mitochondrial dysfunction may contribute to the pathobiology of HPS-related lung complications.

## Supplementary Information

**Additional file 1. Table S1.** qPCR primers used in this study.

## Data Availability

The datasets used and/or analyzed during the current study are available from the corresponding author on reasonable request.

## Abbreviations

HPS-2: Hermansky-Pudlak Syndrome type 2
ETC: Electron transport chain
UPR^mt^: Mitochondrial unfolded protein response
AMPK: 5′-AMP-activated protein kinase
LDHA: Lactic dehydrogenase A
HKII: Hexokinase II
ALDOA: Aldolase A
ROS: Reactive oxygen species
HSP10: Heat shock protein 10
mtHSP70: Mitochondrial heat shock protein 70
HTRA2/OMI: Serine peptidase 2
YME1EL: Yeast mitochondrial AAA metalloprotease.
CLPP: Caseinolytic mitochondrial matrix peptidase proteolytic subunit
LONP1: Long protease 1
SIRT3: Deacetylase sirtuin-3
PGC1: Peroxisome proliferator-activated receptor γ coactivator 1
PRC: Protein Pgc related coactivator
ATP5A: Mitochondrial membrane ATP synthase
COX5A: Cytochrome c oxidase subunit 5a
COX2: Cytochrome c oxidase subunit 2
ATF5: Activating transcription factor 5
ATF4: Activating transcription factor 4
CHOP: C/EBP-homologous protein
NRF1: Nuclear respiration factor 1
TFAM: Mitochondrial transcription factor A
AP3B1: Adaptor related protein complex 3 subunit beta 1
