# Supplementary material for: Hermansky-Pudlak syndrome-2 alters mitochondrial homeostasis in the alveolar epithelium of the lung
Source: Respir Res. 2021 Feb 8;22:49. doi: 10.1186/s12931-021-01640-z (PMC7871590; doi:10.1186/s12931-021-01640-z)
Supplement: Supplementary file 1 — Additional file 1. Table S1. qPCR primers used in this study. [file 12931_2021_1640_MOESM1_ESM.pdf]

Mouse gene	Forward	Reverse
mtND1	CTAGCAGAAACAAACCGGGC	CCGGCTGCGTATTCTACGTT
β-globin	TTGAGACTGTGATTGGCAATGCCT	CCTTTAATGCCCATCCCGGACTT
HKII	GCCAGCCTCTCCTGATTTTAGTGT	GGGAACACAAAAGACCTCTTCTGG
Ldha	GGATGAGCTTGCCCTTGTTGA	GACCAGCTTGGAGTTCGCAGTTA
AldoA	ACATTGCTGAAGCCCAACAT	ACAGGAAAGTGACCCCAGTG
Atf4	AGCAAAACAAGACAGCAGCC	ACTCTCTTCTTCCCCCTTGC
Atf5	CTGGCTCCCTATGAGGTCCTTG	GAGCTGTGAAATCAACTCGCTCAG
Chop	CCTGAGGAGAGAGTGTTCCAG	CCTCTTCGTTTCCTGGGGAT
Hsp10	GGCCCGAGTTCAGAGTCC	TGTCAAAGAGCGGAAGAAACT
mtHsp70	CAAGCGACAGGCTGTCACCAAC	CAACCCAGGCATCACCATTGG
Clpp	TGGGCCCGATTGACGACAGTG	TAGATGGCCAGGCCCGCAGT
Lonp1	CATTGCCTTGAACCCTCTC	ATGTCGCTCAGGTAGATGG
Atp5a	GCTGAGGAATGTTCAAGCAGA	CCAAGTTCAGGGACATACCC
Cox5a	GGGTCACACGAGACAGATGA	ACGACCTCCAAGATGCGAAC
Cox2	ACCTGGTGAACTACGACTGCT	TCCTAGGGAGGGGACTGCTC
Htra2	ATCTCCTTTGCCATCCCTTC	GGTCAGCATCATCACTCCAA
Sirt3	GGATTCGGATGGCGCTTGA	CACCTGTAACACTCCCGGAC
Yme1l1	GCTAAGAAGCAGTTTCGGCT	GAGAGGAATTGTAACCTGAGGC
Pgc1α	GCAGTCGCAACATGCTCAAG	GGGAACCCTTGGGGTCATTT
Tfam	TCCACAGAACAGCTACCCAA	CCACAGGGCTGCAATTTTCC
Nrf1	AGAAACGGAAACGGCCTCAT	CATCCAACGTGGCTCTGAGT
PRC	TGCCTTGCAGTTACTCATGC	CTGACTTGCACTGGCAGGTA
Pgc1β	TGCGGAGACACAGATGAAGA	GGCTTGTATGGAGGTGTGGT
18S	AAACGGCTACCACATCCAAG	CCTCCAATGGATCCTCGTTA

Supplementary Table 1: qPCR primers used in this study.
